# On the Horizon: The Synthetic Opioid U-49900

**DOI:** 10.7759/cureus.1679

**Published:** 2017-09-12

**Authors:** Saeed K Alzghari, Zubair M Amin, Sophie Chau, Steven W Fleming, Kevin Cho, Victor Fung

**Affiliations:** 1 Gulfstream Genomics, Gulfstream Diagnostics; 2 Thomas J. Long School of Pharmacy & Health Sciences, University of the Pacific; 3 School of Pharmacy, Texas Tech University Health Sciences Center; 4 Reference Health Laboratories, Gulfstream Diagnostics; 5 Department of Emergency Medicine, Parkland Health and Hospital System, Dallas Texas

**Keywords:** u-49900, synthetic opioid, drugs of abuse, research chemical, opioids, opioid abuse

## Abstract

Synthetic opioid use continues to be a problem in the United States. New designer opioids continue to be released as "research chemicals" by vendors, leading to widespread use and potentially devastating consequences. U-49900 is a new synthetic opioid with limited clinical data available. Herein, we provide an overview of U-49900, the anecdotal accounts of U-49900 use that clinicians need to be made aware of, and a call for the federal government to take immediate action in curtailing the use of U-49900.

## Editorial

The continued use of opioids continues to rise. In the United States alone, approximately 33,000 people died due to opioid-related deaths in 2015 [1]. Although this statistic accounts for the deaths associated with prescription and illegal opioids, it does not include deaths associated with the use of synthetic opioids, which continue to be developed.

**Figure 1:**
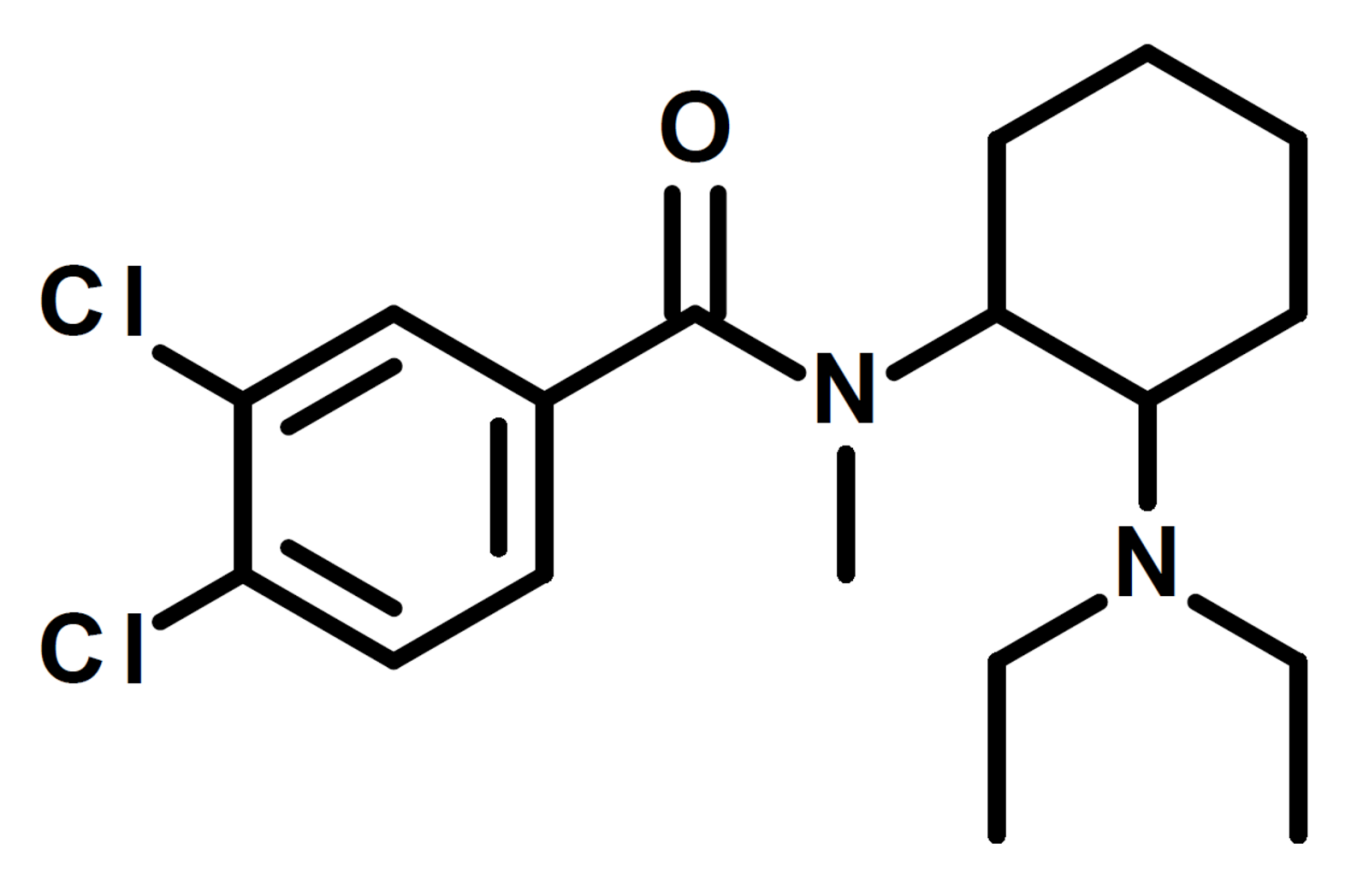
Chemical structure of U-49900

A new synthetic opioid gaining presence is U-49900 (Figure 1). U-49900 is a structural analogue of U-47700, another synthetic opioid recently scheduled by the drug enforcement administration (DEA) to address toxicity-related fatalities as a result of its inappropriate use in the past two years [2]. The Upjohn Company, Michigan, USA, developed both U-47700 and U-49900; however, U-49900 is not scheduled federally [1]. U-49900 never received food and drug administration (FDA) approval, and, presently, no clinical trial data exists for it.

Although preclinical data on rodents exist for U-47700, describing its potency and pharmacodynamics [3], no preclinical data associated with the development of U-49900 exists. Currently, the only published clinical data available on U-49900 is a singular case report from the United States of a 31-year-old male who ingested U-49900 and returned a positive metabolite from a urine specimen; there were no reported signs or symptoms associated with the use of U-49900 [1]. Given that the potency of U-47700 is theorized to be 7.5 times that of morphine and the structural similarities between U-47700 and U-49900, there is cause for concern regarding the risks associated with U-49900 use [1].

Very little is known about U-49900's toxicoepidemiology. A simple online search of “U-49900” retrieved multiple results advertising U-49900 as a “research chemical.” Some of the vendors listed U-49900 as a “hot-selling” item, having an “effect stronger than U-47700,” and of being “top-quality.” Purchasing U-49900 is straightforward; however, this is not surprising since it follows a similar trend to that of other synthetic opioids made readily available for purchase as a supposed “research chemical.”

Interestingly, individuals are willing to take the risk of trying U-49900 and posting about their experience online. The popular website Reddit currently hosts a forum known as “ResearchChemicals” where individuals describe their experiences with synthetic agents [4]. A search for “U-49900” within this particular forum revealed individuals describing little to no effect from U-49900 at doses ranging from 5 mg to 75 mg by intravenous injection, insufflation, or oral ingestion. Furthermore, individuals state that the odor of U-49900 is caustic, it provides no analgesic relief, can cause loss of smell/taste, can cause nerve damage, and can produce a foam-like substance in the lungs [4]. Although these claims are anecdotal in nature, this information can provide clues to clinicians who may encounter patients taking U-49900.

The DEA needs to take a greater role in curtailing access to U-49900 in the United States. To our knowledge, no reported deaths have been associated with U-49900. However, the popularity of this agent will only continue to grow as users seek alternatives to the scheduled U-47700. The DEA needs to crack down on vendors that sell “research chemicals” to limit access. Furthermore, the DEA must seriously take into consideration the multiple online forums in existence dedicated to individuals trying synthetic opioids. The DEA should monitor these websites more actively for trends associated with synthetic opioid use; this will allow the DEA to regulate the inappropriate use of these substances, which can lead to serious harm. The deaths of 46 individuals due to U-47700 could have been avoided had stricter regulations been in place [2]. North Carolina is one of the first states passing a bill to schedule U-49900, and the DEA must take note of their legislation [5]. Worldwide use is documented in Sweden and Spain, showing that U-49900 is establishing an international presence as well [3]. Investing in resources to stop synthetic opioid use at-large by increasing public awareness, providing more clinician education, and scheduling U-49900 at the federal level to limit its sale online will help minimize the spread of U-49900.

## References

[1] Krotulski AJ, Mohr ALA, Papsun DM, Logan BK (2017). Metabolism of novel opioid agonists U-47700 and U-49900 using human liver microsomes with confirmation in authentic urine specimens from drug users. Drug Test Anal.

[2] Drug Enforcement Administration, Department of Justice (2016). Schedules of controlled substances: temporary placement of U-47700 into schedule I. Final order. Fed Regist.

[3] Fabregat-Safont D, Carbón X, Ventura M (2017). Updating the list of known opioids through identification and characterization of the new opioid derivative 3,4-dichloro-N-(2-(diethylamino)cyclohexyl)-N-methylbenzamide (U-49900). Sci Rep.

[4] (2017). Research chemicals. https://www.reddit.com/r/researchchemicals/.

[5] (2017). North Carolina General Assembly House Bill 464/Session Law 2017-115. http://www.ncleg.net/gascripts/BillLookUp/BillLookUp.pl?BillID=H464&Session=2017.

